# Case of the Puerperal Breast—Gaunt

**Published:** 1883-04-20

**Authors:** 


### CARE OF THE PUERPERAL BREAST—GAUNT.

*From American Journal of Obstetrics, October, 1882.*

The cursory treatment of this subject in the text-books justifies the complaint of the author of this paper in his charge against writers on obstetrics of neglecting the subject of every-day interest to the general practitioner, and giving large space to the major operations. All will acknowledge the importance to both mother and child of healthy breasts that perform their functions well, thereby contributing to safe nourishment of the child and developing the material instincts and affections of the mother. You can appreciate the moral suffering of the delicately organized and sensitive mother who is compelled to surrender the charge of her children to the care of another, or what is worse, subject them to the dangers of artificial feeding. The form and minute anatomy of the breast being studied, the author then discusses the puerperal breast with indications for treatment, under the following heads:

1. A deficient secretion of milk.
2. A secretion of impaired quality.
3. An excessive retained secretion.
4. An obstruction to the removal of the fluid.
5. Galactocele.
6. Inflammations.
7. Mammalgia.
8. Affections of the nipple.

And then treats of each of these in detail the cause, progress, prognosis, and treatment:

First. A defective secretion may be due to lack of mammary development, this may be encountered in either extreme of age. Preparatory treatment is suggested during gestation. Avoid excess of fat, and with this in view the physician should correct the popular idea of furnishing the patient with that class of diet which produces excessive fat, at the same time avoid the other extreme, which may sometimes exist from the woman's inability to digest well during gestation.

The general measures for increasing the flow of milk are therefore those constitutional means which have the power of increasing glandular activity and are (a) a dietary presenting an excess of nitrogenous food principles, and (b) systematic exercise regularly taken. Medicines recommended to increase secretion of milk are as fallible as they are numerous. To improve the local tone of the gland skillful massages are strongly urged. This should be regularly employed night and morning. Special attention should be directed to the nipple. They should be rendered prominent and hardened by being pressed between the fingers, and well drawn out. As a medicinal application the following is recommended:

Acid tannic.............................. 4 drachms,
Glycerine.................................. 1 ounce,
Aqua ad.................................... 2 ounces.

This strong solution of tannin acts differently from the weaker combinations of tannin and glycerine, as it actually tans the nipple, making it tough, resolvent and incapable of inflammation.

The first few days after parturition are all important. The milk often diminishes, or ceases entirely during a rise in the mother's temperature, owing to a transient septic poisoning, intestinal disturbance, or other trouble. A permanent injury to the breast may result from neglect of care when the amount of secretion daily is quite irregular.

The writer wisely calls attention to the careless manner in which the infant is nursed. After the secretion is well established, during the first month, he says the infant should be nursed at regular intervals, about every three hours during the day, and double that period at night. Excessive secretion of milk, he says, is a trouble very easily controlled by diet, except where there is an impoverished condition of the milk from relaxation, which causes a passive transudation of the plasm of the blood through the epithelial lining of the acini. For this improve the quality of the milk, and the quantity will diminish in an inverse ratio.

He applies pressure by adhesive plaster, or by a binder, being careful to not compress the nipple. Where it is desirable to stop the secretion entirely, as where the child dies, he uses some compound of lead as the iodide ointment, or strong solution of the acetate.

An obstruction to the removal of milk is a fruitful source of trouble in dealing with the puerperal breast. Then rubbing should be resorted to, or an older and stronger infant applied. Care and skill is needed in the employment of massage.

It is around inflammation of the mammary gland that our especial interest centers. Not only is it a source of considerable mortality from sepsis, but when recovery does take place deformity may result, or the loss of that breast in subsequent pregnacies. It may occur as subcutaneous glandular or subglandular mastitis.

The first object should be to prevent suppuration. The breasts should be well emptied. Careful massage may stimulate the capillary circulation, and diffuse the local induration, or appliances of hot tincture of opium on absorbent cotton may act as a hot fomentation, besides the anodyne to control the pain. If suppuration is inevitable, then the two objects in view should be to hasten the healing process and to prevent scarring. The ung. plumbi iodidi serves a good purpose in many of these cases.

The abuse of the hot poultice in mammary inflammations is as inexcusable as it is general. While there are times that it serves a good purpose, it is evident that its entire omission would be less prolific of trouble than its indiscriminate use. Sulphide of calcium has been largely used to prevent suppuration.

A more careful study of this subject would evidently yield better fruit than the elaboration of Poro's operations, and other subjects of similar magnitude.—*Western Med. Rep.*

